# Large-scale plasma proteomic profiling enhances prediction of heart failure among hypertensive individuals: prospective insights from the UK Biobank

**DOI:** 10.7189/jogh.16.04313

**Published:** 2026-10-19

**Authors:** Maohua Li, Xiao He, Shasha Xiao, Jixiang Wang, Lunchang Wang, Xin Li, Chang Shu, Zhaowei Zhu, Jiehua Li

**Affiliations:** 1 Department of Vascular Surgery, The Second Xiangya Hospital of Central South University, Changsha, China; 2 Molecular Biology Research Center, School of Life Sciences, Hunan Province Key Laboratory of Basic and Applied Hematology, Central South University, Changsha, China; 3 Institute of Vascular Diseases, Central South University, Changsha, China; 4 Center of Vascular Surgery, Fuwai Hospital, National Center for Cardiovascular Disease, Chinese Academy of Medical Sciences and Peking Union Medical College, Beijing, China; 5 Department of Cardiology, The Second Xiangya Hospital of Central South University, Changsha, China

**Keywords:** heart failure, hypertension, plasma proteomics, risk prediction model, protein risk score

## Abstract

**Background:**

Hypertension is a major risk factor for heart failure (HF), but current clinical models have limited ability to identify high-risk individuals before overt disease. Plasma proteomics may capture early molecular changes and improve risk prediction.

**Methods:**

We analysed 16,128 hypertensive UK Biobank Pharma Proteomics Project participants without baseline HF. Plasma levels of 2,911 proteins were measured using the Olink Explore platform. Participants were randomly split 1:1 into training and validation sets. HF-associated proteins were identified using Cox models in the training set, followed by LASSO regression to construct a protein risk score (PRS). Model performance was assessed in the validation set using Harrell’s C-index, time-dependent ROC analysis, Net Reclassification Improvement (NRI), Integrated Discrimination Improvement (IDI), calibration, and bootstrap validation. A parsimonious panel was derived using LightGBM and sequential forward feature selection.

**Results:**

During a median follow-up of 15.1 years, 1,074 participants developed HF. In the training set, 534 proteins were associated with HF after adjustment for demographic and clinical risk factors and were enriched in cytokine signalling, extracellular matrix organisation, and immune-cell migration pathways. The 101-protein PRS was independently associated with incident HF. In the validation set, adding the PRS increased the C-index from 0.670 to 0.786 beyond age and sex, and from 0.727 to 0.787 beyond the Pooled Cohort Equations to Prevent Heart Failure (PCP-HF) model, with improved reclassification and discrimination. PRS levels were elevated years before HF diagnosis. A six-protein panel comprising NT-proBNP, GDF15, BCAN, WFDC2, CTHRC1, and CNTN5 achieved an AUC of 0.775, outperforming NT-proBNP alone.

**Conclusions:**

Plasma proteomic profiling improves HF risk prediction among hypertensive individuals beyond conventional clinical factors and may support earlier risk stratification and precision prevention.

Heart failure (HF) is a major global health issue, affecting over 37 million individuals worldwide, and this number is expected to rise due to an aging population and increasing risk factors [1–3]. Despite therapeutic advances, HF maintains high mortality and hospitalisation rates [4,5]. Hypertension represents the predominant risk factor for HF, driving both disease pathogenesis and progression [6–8]. The Framingham Heart Study revealed 91% of HF patients had antecedent hypertension, and hypertension confers a 2-fold HF risk in men and 3-fold in women, accounting for 39% and 59% of HF cases respectively [9,10].

Hypertension induces a series of maladaptive cardiac changes, including left ventricular hypertrophy (LVH), diastolic dysfunction, and fibrosis, which can ultimately progress to HF, either with reduced ejection fraction (HFrEF) or preserved ejection fraction (HFpEF) [11–13]. The age-standardised prevalence of hypertensive heart disease is estimated to be 217.9 cases per 100,000 people. As patients and populations age, the individual and global disease burden continues to increase [14,15]. Early intervention is critical in reducing morbidity and mortality, yet existing diagnostic and prognostic tools often fail to detect HF at its earliest, subclinical stages in hypertensive individuals.

Traditional risk assessment models relying on clinical and demographic data often fail to accurately predict heart failure onset [16–18]. In contrast, proteomics – the large-scale study of proteins and their functions – has emerged as a promising approach for investigating the molecular mechanisms underlying cardiovascular diseases, including HF [19–21]. Proteomics has provided more precise strategies for the diagnosis, risk assessment, and treatment of HF by identifying novel biomarkers and potential therapeutic targets [22,23]. Research has revealed how HF-related proteins contribute to pathophysiological processes like fibrosis and inflammation, while protein-based scoring models have enhanced prognostic accuracy [24–26]. These advancements have improved personalised HF treatment and management. However, studies investigating the specific plasma proteomic signatures for HF development in hypertensive patients remain scarce, particularly regarding circulating proteins' predictive value for HF risk.

This study investigates the potential of large-scale proteomics to enhance the prediction of heart failure in individuals with hypertension. By integrating proteomic data with established clinical risk factors, we aim to develop more accurate, personalised HF risk models for hypertensive individuals. This approach might lead to more effective prevention and treatment strategies, potentially reducing HF's global burden in this population.

## METHODS

### Study cohort

The UK Biobank (UKB) is a large prospective cohort study examining how genetic, environmental, and lifestyle factors affect long-term health. Between 2006 and 2010, over 500,000 participants aged 37–73 were enrolled across 22 UK assessment centres. This secondary analysis of UK Biobank data follows the Journal of Global Health’s Guidelines for Reporting Analyses of Big Data Repositories Open to the Public (GRABDROP) (Table S1 in the **Online Supplementary Document**) [27]. The reporting checklist is presented in Table S1 of the **Online Supplementary Document**. The UKB study received approval from the Northwest Multi-Center Research Ethics Committee (REC reference numbers: 11/NW/0382, 16/NW/0274, 21/NW/0157) and adhered to the Declaration of Helsinki, with all participants providing written informed consent [28]. All were registered with the National Health Service (NHS) and underwent baseline assessments including questionnaires, physical measurements, biosample collection, and multi-omics profiling (genomics, proteomics, metabolomics). Participants were followed over time to track health outcomes and disease progression [28]. Building on UKB, the UK Biobank Pharma Proteomics Project (UKB-PPP), supported by 13 biopharma companies, focuses on large-scale proteomic analysis of blood samples to aid disease prediction and biomarker discovery [29]. Using the UKB cohort, this study investigates the role of proteomics in predicting heart failure (HF) among hypertensive individuals. To ensure data quality, participants with baseline HF were excluded; only those with hypertension (baseline SBP≥140 mm Hg, DBP≥90 mm Hg, or on antihypertensive medication) and complete proteomic data were included [30]. The final cohort comprised 16,128 participants (59.6% male). With a median follow-up of 15.1 years, 1,074 HF cases occurred. **Figure 1** presents the study flowchart.

**Figure 1:**
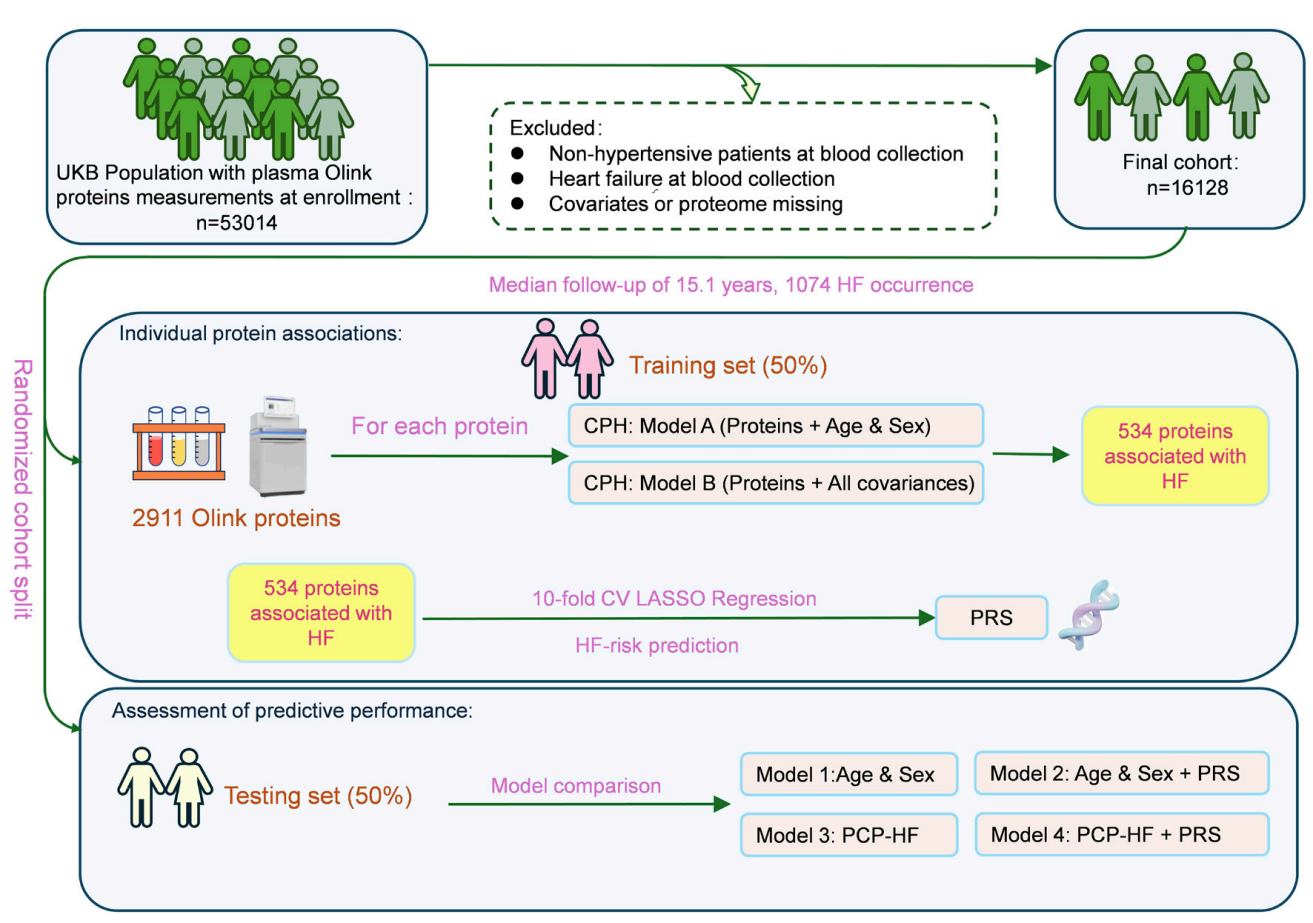
The flowchart of this study. Among 53,014 UK Biobank participants with plasma Olink proteomic measurements, 16,128 hypertensive individuals with complete covariate and proteomic data were included and randomly divided 1:1 into training and validation sets. During a median follow-up of 15.1 years, 1,074 participants developed incident heart failure (HF). In the training set, two Cox proportional hazards models identified 534 HF-associated proteins from 2911 proteins, followed by LASSO Cox regression for feature selection and protein risk score (PRS) development. The PRS was independently evaluated in the validation set and integrated with the Pooled Cohort Equations to Prevent Heart Failure (PCP-HF) model.

### Assessment of covariates

The Pooled Cohort Equations to Prevent Heart Failure (PCP-HF) is a validated 10-year risk prediction model for HF, developed using data from seven community-based cohorts in the USA [31]. It includes a broad range of covariates: sociodemographic factors (age, race, sex); medical history (smoking status, hypertension and diabetes treatment) anthropometric data (systolic blood pressure, body mass index, QRS duration); and biochemical markers (total cholesterol and high-density lipoprotein cholesterol). In this study, nine of the ten PCP-HF covariates were obtained from the UKB data set, with QRS duration excluded due to the absence of electrocardiogram (ECG) data. Previous external validations have demonstrated that the model retains good predictive accuracy without QRS duration [32]. Serum total cholesterol and high-density lipoprotein cholesterol were measured using routine clinical chemistry assays at the UKB central laboratory between November 2015 and October 2017 [33]. Other variables such as age, sex, smoking status, and antidiabetic medication use were collected through standardised questionnaires. BMI was calculated as weight in kilograms divided by height in meters squared. Blood pressure measurements – both systolic and diastolic – were taken twice at separate occasions, with the lower reading used for analysis. Additional details on UKB baseline procedures have been published previously [28].

### Measurements of proteins

This study analysed plasma samples from the UK Biobank (UKB) using the Olink Explore 3,072 proximity extension assay (PEA) technology to quantify 2,941 protein analytes, corresponding to 2,923 unique proteins. These proteins were assessed across eight functional panels – cardiometabolic (I/II), inflammation (I/II), neurology (I/II), and oncology (I/II) – allowing a comprehensive investigation of disease-related molecular pathways [34,35]. Samples were collected during UKB baseline visits from 2007 to 2010, with plasma separated in EDTA anticoagulant tubes and stored at −80°C. The UKB-PPP consortium selected participant subsets *via* a stratified randomisation method, and protein measurements were conducted at Olink’s laboratory in Sweden. Proteomic data are reported as Normalized Protein eXpression (NPX) values, derived from Olink’s DNA-barcoded antibody technology and analysed by high-throughput sequencing on three NovaSeq 6000 systems. A strict quality control process was applied, including baseline sample screening to exclude duplicates, low-volume samples, and withdrawn participants; removal of controls and outliers; sex consistency verification; and batch effect correction through log-transformation of extension control counts and adjustment of plate-specific median NPX values [34]. Proteins were annotated by gene symbols, and standardised NPX values were used without further processing. Further details of proteomic measurement and quality control have been reported previously [34,35].

### Study outcome

The primary endpoint of the study was the incidence of HF, as defined by the International Classification of Diseases, 10th Revision (ICD-10), code I50. HF cases were ascertained through various sources, including death registries, primary care records, hospital admissions, and self-reported diagnoses. The date of onset was determined by the earliest recorded HF event. Participants were monitored from baseline until the first occurrence of HF diagnosis, death, or the end of follow-up on 6 July 2024, whichever came first.

### Development and evaluation of the hypertension-related heart failure (HF) predictive model

Proteins retained by LASSO regression were first entered into an initial Light Gradient Boosting Machine (LGBM) classifier, in which their relative contributions to model performance were quantified according to information gain. To derive a parsimonious predictive protein panel, we then performed sequential forward feature selection by adding proteins individually to the LightGBM model in descending order of importance. This iterative procedure continued until the model reached its maximum area under the receiver operating characteristic curve (AUC). The stopping criterion was defined as the absence of statistically significant AUC improvement in two consecutive DeLong tests after the inclusion of additional proteins. Proteins selected at this optimal point were retained for the final model. SHAP summary plots were subsequently generated to evaluate the relative importance of these proteins and to characterise the direction and magnitude of their contributions to HF risk prediction.

### Statistical analysis

In our analysis, we first performed rigorous quality control on the proteomic data by excluding proteins with more than 20% missing values across samples, leaving 2,911 proteins for subsequent analyses. Remaining missing data were imputed using the K-nearest neighbors (KNN) method with K = 5 to ensure completeness. The study population was then randomly divided equally into a training set (50%) and a validation set (50%). Within the training set, we applied multivariable Cox proportional hazards models to evaluate the association between each protein and HF risk, developing two models: Model A adjusted for age and sex to account for basic demographic factors, and Model B further adjusted for smoking status, ethnicity, HbA1c, BMI, eGFR, total cholesterol, HDL, LDL, use of anti-hyperglycemic and anti-hypertensive medications, as well as systolic and diastolic blood pressure. To control for multiple testing, the Benjamini-Hochberg (BH) correction was applied, with significant results presented in a volcano plot. Proteins that were statistically significant in both models underwent functional enrichment analyses including Gene Ontology (GO) and Kyoto Encyclopedia of Genes and Genomes (KEGG) to investigate their biological functions (using the R package clusterProfiler). Feature selection was then performed using Least Absolute Shrinkage and Selection Operator (LASSO) regression with 10-fold cross-validation to pinpoint the most predictive proteins. A protein risk score (PRS) was constructed based on the proteins selected by LASSO, using the formula:



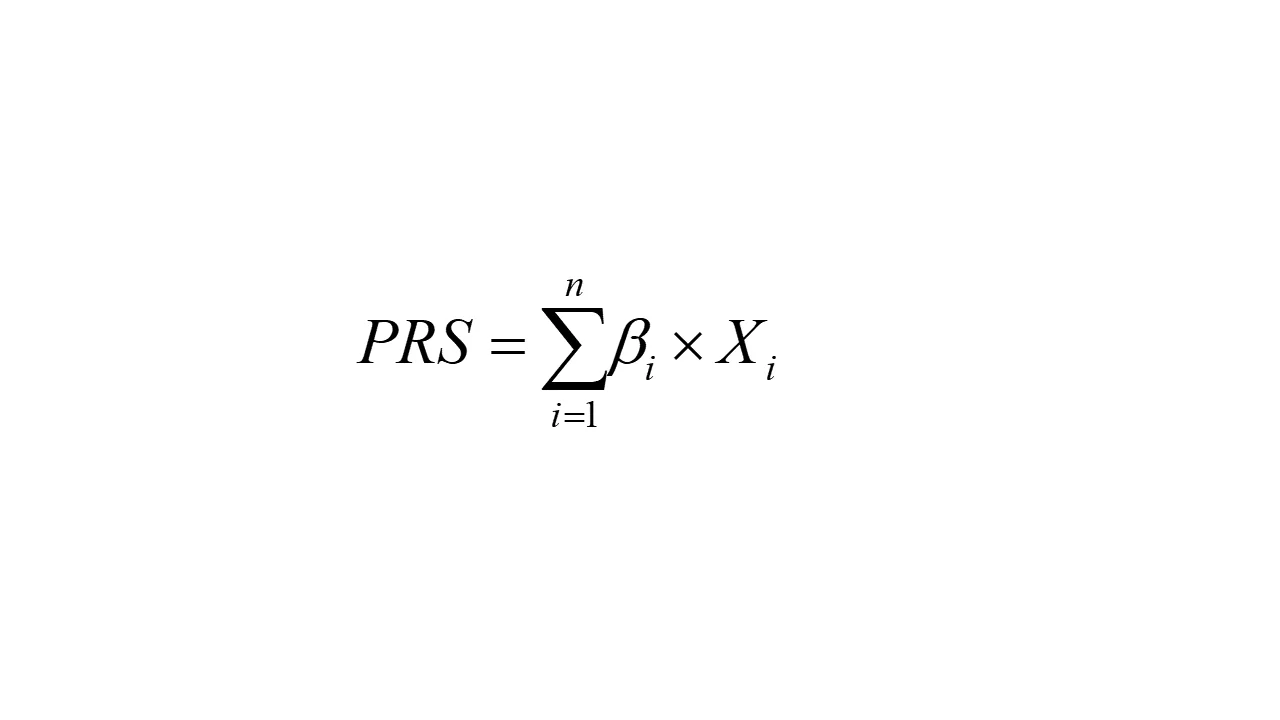



where βᵢ denotes the coefficient for protein i, and Xᵢ represents its corresponding NPX expression value [36,37]. Based on this approach, we developed four predictive models: Model 1 included age and sex; Model 2 combined age, sex, and the protein risk score (PRS); Model 3 was the PCP-HF model; and Model 4 incorporated the PCP-HF model plus the PRS. The performance of these models was assessed using Harrell’s C-index and the area under the receiver operating characteristic (ROC) curve. To ensure reliability, 1,000 bootstrap resampling iterations were performed to estimate 95% confidence intervals (CIs). Furthermore, improvements in model discrimination were quantified using the continuous net reclassification index (NRI) and integrated discrimination improvement (IDI), calculated *via* the R package survIDINRI over 1,000 perturbed training iterations. The cohort was first randomly divided into training and testing sets at a 1:1 ratio. All feature-screening procedures, including Cox proportional hazards analyses and subsequent LASSO regression, were conducted exclusively within the training set. The testing set was reserved solely for independent validation. To explore the temporal relationship between baseline proteomic risk and subsequent HF onset, we performed a retrospective time-aligned analysis using single baseline PRS measurements. HF cases were categorised according to the interval between baseline assessment and incident HF diagnosis. For each HF case, five controls without incident HF during follow-up were randomly matched by age and sex. Baseline PRS distributions were then compared across different pre-diagnostic time windows. As proteomic profiling was performed only once at baseline, this analysis does not represent longitudinal within-individual proteomic trajectories, but rather evaluates whether elevated baseline PRS can be detected years before clinical HF onset [38]. All statistical analyses were carried out in *R* version 4.3.2 (R Foundation for Statistical Computing, Vienna, Austria).

## RESULTS

### Characteristics of participants

The study included 16,128 hypertensive participants with a mean age of 61 years, of whom 59.63% were male. Over a median follow-up of 15.1 years, 1074 individuals (6.66%) developed heart failure (HF). Compared to those without HF, participants who developed the condition were generally older, more often male and current smokers, and exhibited higher HbA1c levels, lower high-density lipoprotein (HDL), and higher low-density lipoprotein (LDL) levels (**Table 1**).

**Table 1:** Population characteristics at baseline and incident endpoints

Characteristics	Whole cohort (n = 16,128)	Incident HFs	
		**Yes (n = 1074)**	**No (n = 15,054)**
Age*	61 (55–65)	65 (60–67)	61 (54–65)
Sex*			
*Male*	9,617 (59.63%)	773 (71.97%)	8,844 (58.75%)
*Female*	6,511 (40.37%)	301 (28.03%)	6,210 (41.25%)
Ethnicity			
*Black*	207 (1.28%)	9 (0.84%)	198 (1.32%)
*White*	15,259 (94.61%)	1,017 (94.69%)	14,242 (94.61%)
BMI*	27.87 (25.27, 31.01)	29.33 (26.27, 32.93)	27.79 (25.22, 30.86)
Systolic blood pressure	148 (142, 157)	147 (138, 159)	148 (142, 157)
Diastolic blood pressure*	87 (80, 93)	83 (75, 90)	87 (81, 93)
Glycated haemoglobin*	36 (33.4, 39)	37.7 (34.5, 41.9)	35.9 (33.3, 38.8)
Total cholesterol*	5.60 (4.76, 6.45)	5.04 (4.22, 5.98)	5.64 (4.81, 6.47)
HDL*	1.35 (1.12, 1.62)	1.24 (1.03, 1.52)	1.36 (1.13, 1.63)
LDL*	3.49 (2.85, 4.14)	3.09 (2.50, 3.81)	3.51 (2.88, 4.16)
eGFR*	92.87 (87.85, 98.44)	89.46 (83.40, 94.83)	93.08 (88.11, 98.62)
Anti-hyperglycaemic medication*	206 (1.28%)	40 (3.72%)	166 (1.1%)
Anti-hypertensive medication*	4,619 (28.64%)	539 (50.19%)	4,080 (27.1%)
Currently smoking*	1,559 (9.67%)	159 (14.8%)	1,400 (9.3%)

BMI – body mass index, HDL – high-density lipoprotein, LDL – low-density lipoprotein, eGFR – estimated glomerular filtration rate

*Values are given as median (interquartile range), or n (%). Mann-Whitney U for continuous variables, χ^2^ for categorical variables; Benjamini–Hochberg correction for multiple testing. *P* < 0.01.

### Associations of proteins with heart failure (HF) risk

The entire cohort was randomly split into training and testing sets at a 1:1 ratio, with baseline characteristics comparable between the two groups (Table S2 in the **Online Supplementary Document**). In the training data, out of the 2,911 plasma proteins analysed, 1,084 showed significant associations with heart failure (HF) in Model A, which adjusted for age and sex, after Benjamini-Hochberg (BH) correction (**Figure 2**, Panel A; Table S3 in the **Online Supplementary Document**). In Model B, which further accounted for smoking, race, HbA1c, body mass index (BMI), eGFR, total cholesterol, HDL, LDL, use of hypoglycemic and antihypertensive medications, as well as systolic and diastolic blood pressure, 553 proteins remained significantly associated with HF (**Figure 2**, Panel B; Table S4 in the **Online Supplementary Document**). A Venn diagram revealed 534 proteins consistently linked to HF across both models (**Figure 2**, Panel C). Among these proteins, NT-proBNP exhibited the most significant association with HF in both models (Model A: HR = 1.680, *P* = 2.20 × 10^−67^; Model B: HR = 1.610, *P* = 6.61 × 10^−47^). Other notable proteins significantly linked to HF included: NPPB (coding for BNP) (Model A: HR = 1.410, *P* = 4.07 × 10^−32^; Model B: HR = 1.327, *P* = 1.21 × 10^−19^), WFDC2 (Model A: HR = 2.634, *P* = 5.54 × 10^−50^; Model B: HR = 2.721, *P* = 6.80 × 10^−27^), GDF15 (Model A: HR = 2.545, *P* = 2.42 × 10^−57^; Model B: HR = 2.047, *P* = 1.37 × 10^−19^). These results suggest that elevated levels of NT-proBNP, NPPB, WFDC2 and GDF15 are associated with an increased risk of HF. Conversely, several proteins with hazard ratios (HRs) less than 1 – indicating a reduced risk of HF – were identified. These included: ENPP5 (Model A: HR = 0.598, *P* = 3.77 × 10^−13^; Model B: HR = 0.637, *P* = 1.44 × 10^−8^), LRRN1 (Model A: HR = 0.500, *P* = 1.71 × 10^−11^; Model B: HR = 0.609, *P* = 1.66 × 10^−5^), BCAN (Model A: HR = 0.413, *P* = 3.29 × 10^−15^; Model B: HR = 0.577, *P* = 2.40 × 10^−5^), CTSV (Model A: HR = 0.549, *P* = 3.23 × 10^−8^; Model B: HR = 0.610, *P* = 3.00 × 10^−5^). Functional enrichment analyses, including GO and KEGG, performed on proteins significantly associated with HF in both models, revealed enrichment in pathways such as cytokine-cytokine receptor interaction, cytokine activity, collagen-containing extracellular matrix, and myeloid leukocyte migration, among others (**Figure 2**, Panel D; Table S5 in the **Online Supplementary Document**). After excluding participants who developed HF during the first 2 years of follow-up, the sensitivity analysis produced results that were highly concordant with the primary analysis. Protein effect estimates showed an almost perfect correlation with the original findings (r = 0.99, *P* < 2.2 × 10^−16^), supporting the robustness of the associations and suggesting that they were unlikely to be explained by reverse causality (Figure S1 in the **Online Supplementary Document**).

**Figure 2:**
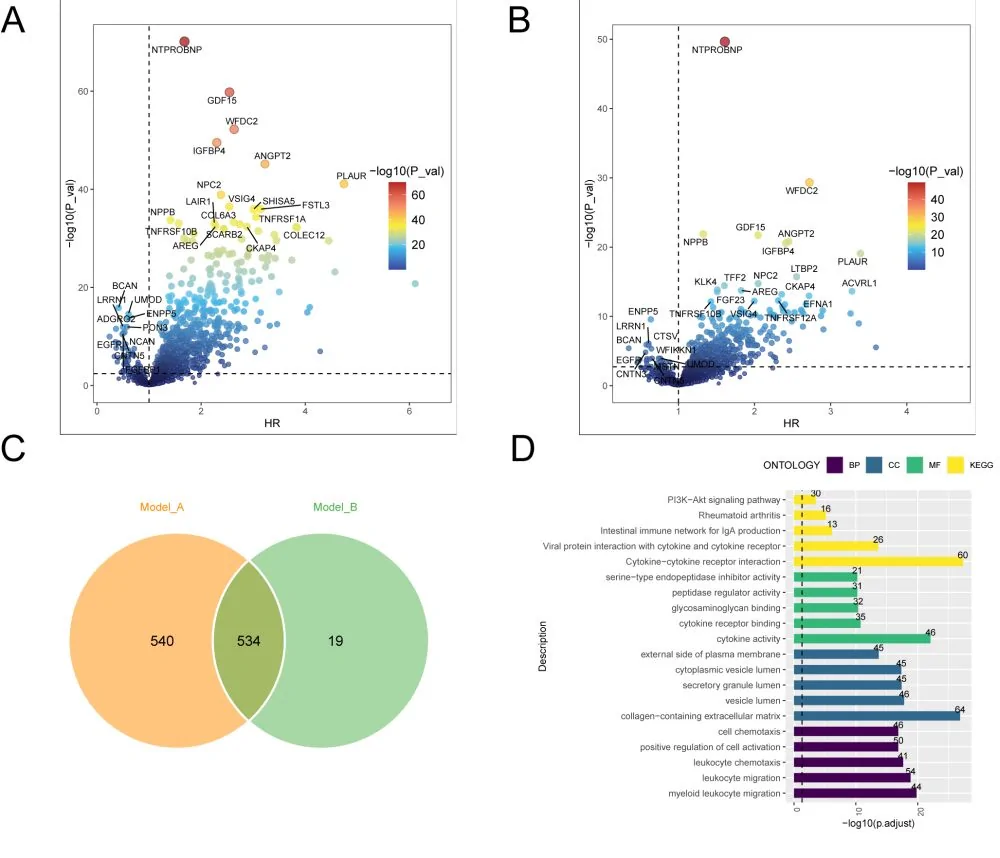
Associations of plasma proteins with heart failure (HF). (Panel A) The volcano plot shows the overall association of 2,911 proteins with HF in Model A, with HR (x-axis) and −log10 (*P*-value) (y-axis). Proteins above the horizontal black dashed line are significantly associated with HF after BH correction. (Panel B) The volcano plot shows the overall association of 2911 proteins with HF in Model B, with HR (x-axis) and −log10 (*P*-value) (y-axis). Proteins above the horizontal black dashed line are significantly associated with HF after BH correction. (Panel C) The Venn diagram shows the proteins significantly associated with HF in both Model A and Model B. (Panel D) Enrichment analysis for Gene Ontology (GO) and the Kyoto Encyclopedia of Genes and Genomes (KEGG) was performed using the R package clusterProfiler for the significant proteins in both Model A and Model B. Statistical significance was defined as adjust. *P*-value <0.05 (vertical dashed line). The number next to each bar represents the number of proteins observed in each pathway. BP – biological process, CC – cellular component, MF – molecular function.

### Performance of predictive models

Using the training set, LASSO regression selected 101 proteins by minimising the partial likelihood deviance (Figure S2 and Table S6 in the **Online Supplementary Document**). A protein risk score (PRS) was then calculated for the hypertensive population as a weighted sum of 101 selected proteins. In the training set, after adjusting for age and sex, the PRS was significantly associated with an increased risk of HF events (hazard ratio (HR) = 3.06; 95% confidence interval (CI) = 2.84, 3.30, *P* = 5.36 × 10^−191^). This association remained statistically significant even after additional adjustment for PCP-HF risk factors (HR = 2.99; 95% CI = 2.75, 3.25, *P* = 1.92 × 10^−146^). Similar results were observed in the testing set (Table S7 in the **Online Supplementary Document**). Model performance assessed in the testing cohort showed that models incorporating the plasma PRS consistently outperformed their counterparts without PRS across various risk thresholds, as demonstrated by time-dependent ROC curve analysis (Figure S3 in the **Online Supplementary Document**). Further evaluation of performance metrics highlighted that models incorporating PRS exhibited enhanced discriminative ability: age & sex *vs.* age & sex + PRS: ΔC = 0.116 (95% CI = 0.092, 0.140), net reclassification improvement (NRI) = 0.388 (95% CI = 0.332, 0.439). PCP-HF *vs.* PCP-HF + PRS: ΔC = 0.060 (95% CI = 0.040, 0.078), NRI = 0.333 (95% CI = 0.276, 0.397), IDI = 0.079 (95% CI = 0.059, 0.105) (**Table 2**, **Table 3**). These improvements were further supported by consistent increases in integrated discrimination improvement (IDI) with PRS inclusion (**Table 3**). However, when compared to the model age & sex + PRS, the comprehensive PCP-HF model showed almost no improvement in reclassification, with: ΔC = −0.060 (95% CI = −0.079, −0.041); NRI = −0.240 (95% CI = −0.314, −0.156); IDI = −0.073 (95% CI = −0.096, −0.048). Comparable model performances were observed in the training cohort (Tables S8 and S9 in the **Online Supplementary Document**). To evaluate the possibility of model overfitting, we additionally conducted bootstrap optimism correction using 1,000 resampling iterations. Across all prediction models, the degree of optimism was negligible, as shown by the close agreement between the apparent and optimism-corrected C-indices. For example, the C-index changed only from 0.820 to 0.819 for the Age + Sex + PRS model and from 0.824 to 0.821 for the PCP-HF+PRS model. These findings suggest that the predictive performance was robust and stable (Table S10 in the **Online Supplementary Document**). Calibration plots demonstrated improved agreement between predicted and observed 10-year HF risk after incorporation of the PRS, suggesting enhanced calibration of the prediction models (Figure S4 in the **Online Supplementary Document**).

**Table 2:** Internal model validation of test set: absolute discriminative capacities*

Metric	Age & sex	Age & sex + PRS	PCP-HF	PCP-HF + PRS
Harrell’s C	0.670 (0.648, 0.693)	0.786 (0.766, 0.805)	0.727 (0.705, 0.747)	0.787 (0.767, 0.807)
Sensitivity	0.771 (0.642, 0.838)	0.642 (0.564, 0.756)	0.694 (0.566, 0.770)	0.664 (0.601, 0.717)
Specificity	0.490 (0.435, 0.620)	0.791 (0.677, 0.863)	0.645 (0.586, 0.766)	0.777 (0.702, 0.832)

PCP-HF – pooled cohort equations to prevent heart failure, PRS – protein risk score

*Model performance is assessed in the validation partition. Values are Harrell’s concordance indices, sensitivities, and specificities with 95% confidence intervals in parentheses; the intervals were obtained from 1000 bootstrap resamples of the validation partition.

**Table 3:** Internal model validation of test set: metrics of relative performance*

Metric	Age & sex *vs.* age & sex + PRS	PCP-HF *vs.* PCP-HF + PRS	Age & sex *vs. *PCP-HF	Age & sex + PRS *vs.* PCP-HF	Age & sex *vs.* PCP-HF + PRS	Age & sex + PRS *vs.* PCP-HF + PRS
△C	0.116 (0.092, 0.140)	0.060 (0.040, 0.078)	0.056 (0.041, 0.072)	−0.060 (−0.079, −0.041)	0.117 (0.093, 0.138)	0.001 (−0.003, 0.004)
NRI	0.388 (0.332, 0.439)	0.333 (0.276, 0.397)	0.265 (0.212, 0.330)	−0.240 (−0.314, −0.156)	0.404(0.352, 0.462)	0.143 (0.074, 0.222)
IDI	0.099 (0.077, 0.127)	0.079 (0.059, 0.105)	0.026 (0.019, 0.040)	−0.073 (−0.096, −0.048)	0.105(0.083, 0.137)	0.006 (0.002, 0.016)

NRI – net reclassification improvement, IDI – integrated discrimination improvement, PCP-HF – pooled cohort equations to prevent heart failure, ΔC – difference in Harrell’s C-index

*Values are differences between the second and first model named in each column, followed by 95% confidence intervals in parentheses.

### Protein risk score and the risk of clinical progression

We then assessed the prognostic value of the baseline plasma protein risk score (PRS) for incident heart failure (HF). Participants were stratified into high- and low-risk groups using the surv_cutpoint function in the R package survminer (version 0.4.9; CRAN). This function identified the cutoff that best separated participants who developed HF from those who remained free of HF during follow-up. Elevated baseline plasma PRS was strongly associated with a higher risk of HF development (Figure S5, Panel A in the **Online Supplementary Document**). Additionally, a retrospective time-scale analysis examined baseline PRS distributions across different pre-diagnostic intervals relative to HF diagnosis, comparing these changes to those in non-HF controls over the same period. HF patients exhibited significantly elevated PRS levels as early as 16 years prior to diagnosis, with values increasing progressively up to disease onset (Figure S5, Panel B in the **Online Supplementary Document**).

### Light Gradient Boosting Machine (LightGBM) machine learning selects the best combination of predictive proteins

To improve clinical applicability and reduce model complexity, we further refined the 101 proteins selected by LASSO using a Light Gradient Boosting Machine (LightGBM) framework combined with sequential forward feature selection. Candidate proteins were ranked according to their contribution to predictive performance based on information gain (Table S11 in the **Online Supplementary Document**). NT-proBNP and GDF15 exhibited the highest feature importance scores, and model discrimination improved rapidly as top-ranking proteins were sequentially incorporated, reaching a plateau after the inclusion of six proteins (**Figure 3**, Panel A). Accordingly, a parsimonious six-protein panel consisting of NT-proBNP, GDF15, BCAN, WFDC2, CTHRC1, and CNTN5 was selected for subsequent analyses. To enhance model interpretability, Shapley additive explanation (SHAP) analysis was performed to quantify the contribution and directionality of each protein to HF risk prediction (**Figure 3**, Panel B). The six proteins contributed to predicted HF risk in different directions, with NT-proBNP making the largest contribution to the model. Given the established role of NT-proBNP as a clinical biomarker of HF and its dominant predictive contribution within the model, we further compared its predictive performance with that of the multi-protein panel. In 10-fold cross-validation analyses, NT-proBNP alone achieved an AUC of 0.685, whereas the six-protein panel significantly improved discrimination (AUC = 0.775; DeLong test, *P* < 0.001). The demographic model alone yielded an AUC of 0.736. Notably, integration of the six-protein panel with the conventional PCP-HF risk model resulted in the highest predictive performance (AUC = 0.781; DeLong test, *P* < 0.001), highlighting the complementary value of proteomic signatures when combined with established clinical risk factors (**Figure 3**, Panel C).

**Figure 3:**
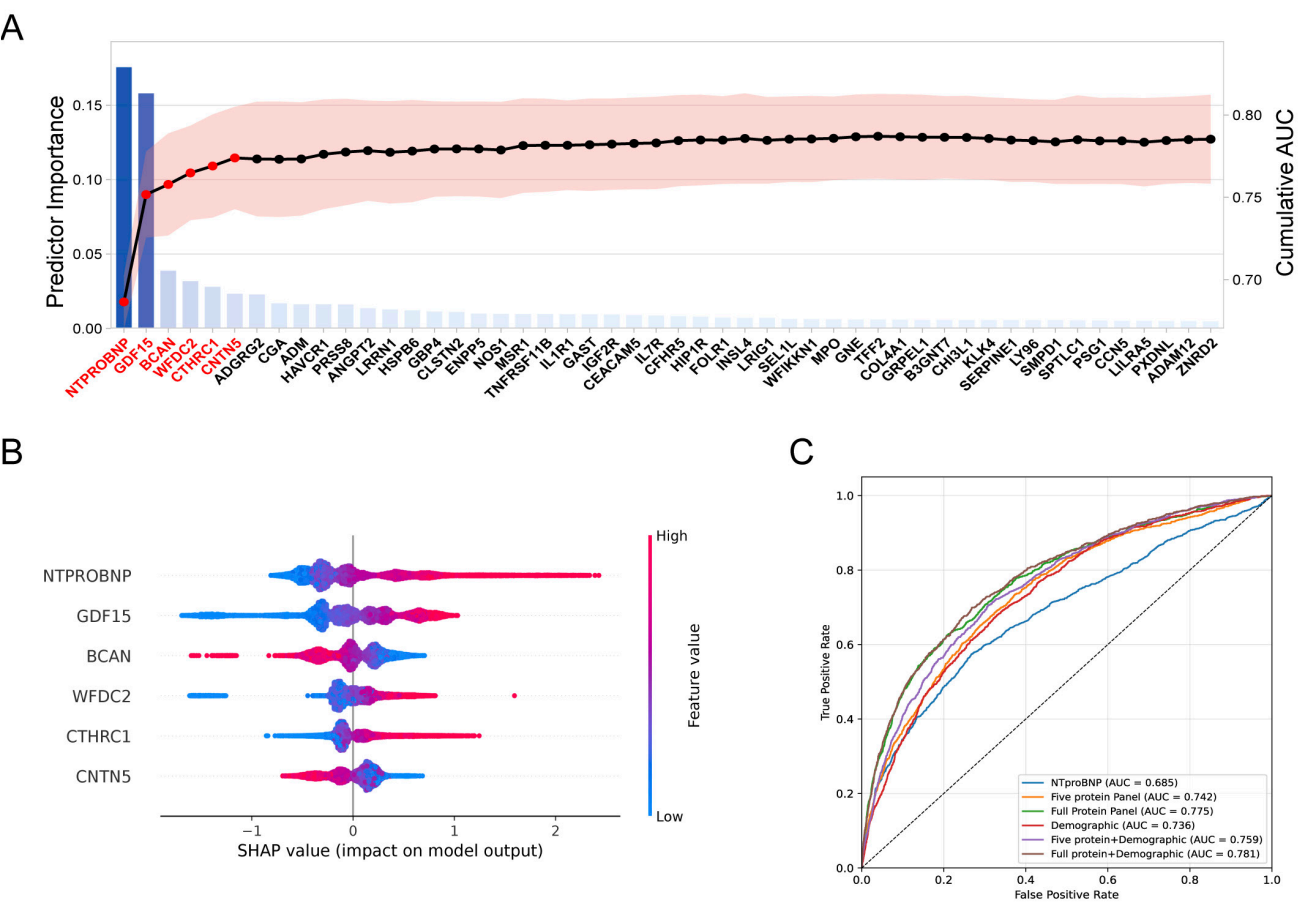
Predictor selection, feature importance, and ROC curves for incident heart failure (HF) prediction in individuals with hypertension. Panel A. Bar plot showing the relative importance of LASSO-selected proteins for predicting incident HF among hypertensive participants. The line plot on the right axis shows the cumulative AUC as proteins were sequentially added through forward selection. Shaded areas represent 95% confidence intervals from 10-fold cross-validation. Proteins highlighted in red indicate the final optimal panel, defined as the point after which two consecutive DeLong tests showed no significant improvement in model performance (two-sided *P* > 0.05). Panel B. SHAP summary plot displaying the contribution of each selected predictor to HF risk estimation. Wider horizontal dispersion indicates greater predictive impact. Color represents protein expression level, ranging from low (blue) to high (red), and the x-axis direction reflects lower *vs.* higher predicted HF risk. Panel C. ROC curves comparing the predictive performance of different models for incident HF in hypertensive individuals.

## DISCUSSION

In this study, we demonstrated that large-scale plasma proteomics substantially improves the prediction of incident heart failure (HF) among individuals with hypertension. Utilising the UK Biobank Pharma Proteomics Project (UKB-PPP) cohort, which included over 16,000 hypertensive participants without baseline HF and a median follow-up exceeding 15 years, we integrated plasma proteomic data with traditional clinical risk factors to develop and validate a robust proteomic risk score (PRS) that markedly enhances HF risk stratification. Our results show that proteomics reveals important biological mechanisms underlying HF development beyond traditional clinical parameters, reflecting the complex pathophysiology of HF in hypertensive patients. The study thereby highlights the promise of large-scale proteomic technologies to identify predictive biomarkers, improve personalised risk prediction models, and potentially guide targeted preventative or therapeutic strategies.

Hypertension is a major risk factor for the development of HF, especially HF with preserved ejection fraction (HFpEF) [39,40]. Chronic elevated blood pressure induces structural and functional cardiac changes, such as left ventricular hypertrophy, fibrosis, and diastolic dysfunction, ultimately impairing cardiac function [41,42]. Epidemiological studies indicate a significant burden of hypertensive heart disease, with rising prevalence driven by global population aging [43,44]. The transition from hypertension to HF is a complex process involving multiple pathways such as vascular remodeling, inflammation, and neurohormonal activation [45]. Clinical trials, including the SPRINT trial, have shown that intensive blood pressure control reduces the risk of HF onset [46,47]. However, despite effective hypertension treatment, many patients still develop HF, underscoring the need for better risk stratification. Recent advances in proteomics have uncovered novel biomarkers associated with a series of cardiovascular events, offering potential for earlier detection and personalised management [48–50]. Large-scale plasma proteomics can capture biological processes underlying hypertensive heart disease, such as inflammation and myocardial fibrosis, enhancing predictive models beyond traditional risk factors. These insights are guiding emerging preventive and therapeutic strategies targeting the pathophysiological mechanisms in hypertensive HF. Overall, integrating clinical, proteomic, and molecular data holds promise for reducing the global heart failure burden attributable to hypertension through precision prevention and treatment.

Our study builds upon and extends prior research demonstrating the role of plasma proteomics in cardiovascular disease risk assessment. Proteomics provides a high-resolution, systems-level view of circulating proteins, revealing pathophysiological insights beyond traditional risk factors or genomics [34,51,52]. Previous studies have shown proteomics' predictive value for cardiovascular outcomes, but typically in broadly defined cohorts. Henry et al. identified 44 circulating proteins associated with incident heart failure using large-scale plasma proteomics and Mendelian randomisation, of which eight showed robust causal relationships and seven are druggable targets [53]. Similarly, Ramonfaur *et al*. linked proteomic profiles to frailty and HF risk in aged populations using large-scale proteomics [54]. Our study focuses specifically on hypertensive individuals, a subgroup particularly vulnerable to HF but underserved by current predictive models. The cohesive picture emerging from multi-omics and proteomics studies suggests that personalised HF prediction is feasible and likely to improve clinical outcomes.

Among the proteins contributing most strongly to improved prediction accuracy, several have well-established roles in HF pathogenesis. Natriuretic peptides such as NT-proBNP and NPPB (coding for BNP), already standard as HF biomarkers [55–57], were also identified among predictive proteins, serving as internal validation of our results. Whey acidic protein four-disulfide core domain 2 (WFDC2), also known as HE4, is primarily studied in ovarian and prostate cancers, which regulates cell signaling and immune responses [58,59]. While its HF role is unclear, WFDC2 may promote cardiac remodeling/fibrosis *via* EGFR/AKT pathways. Further studies are needed to explore its utility as a biomarker or therapeutic target in HF. Growth differentiation factor-15 (GDF-15), a marker of inflammation and cellular stress, is strongly associated with cardiac remodeling and adverse outcomes in HF [60,61]. Beyond these, our analysis highlighted additional proteins involved in metabolic regulation, extracellular matrix turnover, and immune responses, suggesting multiple convergent pathways contribute to HF progression in hypertensive individuals. Inflammation/fibrosis markers align with hypertensive heart disease mechanisms, where chronic pressure overload causes myocardial hypertrophy, fibrosis, and cardiac dysfunction. The identification of proteins linked to metabolic dysregulation aligns with the high prevalence of metabolic syndrome features within hypertensive populations and their recognised role in HF pathogenesis. This biologically plausible landscape of proteomic markers supports the clinical relevance and potential utility of incorporating such markers in risk prediction models.

The enhanced predictive accuracy of proteomic-based models compared to traditional clinical risk factors is an important advance. Classic risk scores primarily rely on demographic and clinical variables such as age, blood pressure levels, comorbidities, and biochemical markers like cholesterol; however, their capacity to identify which hypertensive patients will progress to HF remains limited [62–64]. By integrating proteomic data, our model captures subclinical biological processes preceding overt disease, allowing for earlier and more sensitive detection of high-risk individuals. The net reclassification improvement and higher C-statistics observed in our evaluation confirm the added value of proteomics. These findings resonate with some other recent investigations. For example, Dubin *et al.* identified novel circulating proteins associated with incident heart failure in chronic kidney disease patients using large-scale proteomics and developed proteomic risk models that outperform existing prediction tools [48]. And Chan *et al.* integrated large-scale plasma proteomics with single-cell cardiac transcriptomics to identify and prioritise novel and established protein biomarkers associated with heart failure following myocardial infarction [65]. Our study focuses on hypertension, emphasising the need for tailored approaches in this large, at-risk subgroup. Given the heterogeneity in hypertensive patients-ranging from well-controlled individuals to those with multiple metabolic comorbidities – proteomics provides enhanced risk stratification unattainable with clinical variables alone.

This study benefits from several notable strengths. The sample size of over 16,000 hypertensive individuals, coupled with prolonged follow-up exceeding 15 years, provides strong statistical power and temporal depth for incident HF prediction. The rigorous exclusion of participants with HF at baseline reduces bias from pre-existing disease and enhances the interpretability of proteomic predictors as true risk markers rather than consequences of disease. Furthermore, the UKB-PPP platform’s highly standardised and high-throughput proteomic assay allows for comprehensive and reproducible measurement of thousands of plasma proteins, facilitating discovery of novel markers and development of risk models. Our approach of integrating proteomic data with established clinical factors reflects a practical clinical scenario, underpinning translational potential.

Nonetheless, several limitations should be considered. First, the study population was derived from the UK Biobank, whose participants are generally healthier, less socioeconomically deprived, and less ethnically diverse than the general population; because the cohort was predominantly of European ancestry, the generalisability of the protein risk score to other ethnic groups and healthcare settings requires external validation. Second, although we adjusted for multiple clinical covariates, residual confounding cannot be fully excluded, as factors such as lifestyle, medication adherence, socioeconomic status, hypertension duration, treatment intensity, and hypertensive target-organ damage may influence both proteomic profiles and HF risk but were not completely captured. Third, proteomic measurements were obtained from a predefined Olink panel at a single baseline time point; therefore, some relevant low-abundance proteins, post-translational modifications, or temporal changes in protein expression may have been missed, and serial proteomic profiling may further improve risk prediction and mechanistic interpretation. Fourth, HF outcomes were identified using ICD-based records without systematic differentiation between HFrEF and HFpEF. Given the strong link between hypertension and HFpEF, future studies incorporating echocardiographic or imaging data are needed to explore subtype-specific proteomic signatures. In addition, the PCP-HF model was adapted because QRS duration was unavailable, which may affect calibration and comparability with the original model.

The findings hold meaningful implications for clinical practice and future research. The proteomic risk score enhances early identification of high-risk hypertensive individuals, enabling personalised monitoring and timely use of cardioprotective therapies like SGLT2 inhibitors. Identified proteins such as GDF-15 and WFDC2 may serve as therapeutic targets or markers for myocardial remodeling. Future research should validate this score in diverse cohorts, integrate it with genomics and metabolomics for deeper insights, and develop cost-effective assays for clinical use. Prospective trials are essential to assess whether proteomic-guided strategies improve patient outcomes, while functional studies of associated proteins could inform novel treatments.

## CONCLUSIONS

In conclusion, this study demonstrates that large-scale plasma proteomics significantly enhances heart failure (HF) risk prediction in hypertensive individuals beyond conventional clinical risk factors. The identified proteomic signatures capture key pathophysiological mechanisms underlying HF development, offering improved risk stratification in this high-risk population. These findings support the integration of proteomic biomarkers into clinical practice, enabling precision medicine strategies for early identification, risk management, and potential therapeutic targeting in hypertension-associated HF.

## Additional material


Online Supplementary Document

## Data Availability

**Data availability:** Data from the UK Biobank are available to bona fide researchers upon application through the UK Biobank Access Management System (https://www.ukbiobank.ac.uk/enable-your-research/apply-for-access). Other data supporting the findings of this study are available from the corresponding author upon reasonable request.
