# Supplementary material for: Large-scale plasma proteomic profiling enhances prediction of heart failure among hypertensive individuals: prospective insights from the UK Biobank
Source: J Glob Health. 2026 Oct 19;16:04313. doi: 10.7189/jogh.16.04313 (PMC13688282; doi:10.7189/jogh.16.04313)
Supplement: Supplementary file 1 [file jogh-16-04313-s001.zip › jogh-16-04313-s001.docx]

**Supplement to: Li M, He X, Xiao S, Wang J, Wang L, Li X, et al. Large-scale plasma proteomic profiling enhances prediction of heart failure among hypertensive individuals: prospective insights from the UK Biobank. J Glob Health. 2026;16:04313.**

**Supplementary Figures S1-5**

**Figure S1.** Sensitivity analysis excluding participants who developed heart failure (HF) within the first 2 years of follow-up. Scatter plot showing the correlation of protein effect estimates between the primary analysis and the sensitivity analysis. (A) Age- and sex-adjusted model.(B) Fully adjusted model.

**Figure S2.** Feature selection using LASSO regression. (A, B) Lasso coefficient path plot and cross-validation curve for lasso regression analysis.

**Figure S3.** Receiver-operating characteristic (ROC) curve. Receiver-operating characteristic (ROC) curve illustrate discriminative performance of the different models. PCP-HF, Pooled Cohort Equations to Prevent Heart Failure.

**Figure S4.** Calibration plots for the four prediction models in the testing cohort. (A)Age + Sex; (B)Age + Sex + PRS; (C) PCP-HF model; (D) PCP-HF + PRS model.

**Figure S5.** Predictive performance of baseline protein risk score for clinical progression risk. (A) Kaplan–Meier curves present the clinical progression to HF over time, visualized for individuals with low (blue line) and high (red line) baseline protein risk score. (B) The dynamic changes protein risk score before the diagnosis of HF were plotted. A nested case-control study was conducted, where an individual with an incident event was matched with five healthy controls based on matching criteria of age and sex.
