# Supplementary material for: Large-scale plasma proteomic profiling enhances prediction of heart failure among hypertensive individuals: prospective insights from the UK Biobank
Source: J Glob Health. 2026 Oct 19;16:04313. doi: 10.7189/jogh.16.04313 (PMC13688282; doi:10.7189/jogh.16.04313)
Supplement: Supplementary file 1 [file jogh-16-04313-s001.zip › jogh-16-04313-s002.docx]

Supplementary Table1 Population characteristics of training and test set.

| **Characteristics** | **Whole cohort**  **(n=** **16,128)** | **Training set**  **(n=** **8,064)** | **Test set**  **(n=** **8,064)** |
| --- | --- | --- | --- |
| Age | 61(55-65) | 61(55-65) | 61(55-65) |
| Sex |  |  |  |
| Male | 9617(59.63%) | 4794(59.45%) | 4823(59.81%) |
| Female | 6511(40.37%) | 3270(40.55%) | 3241(40.19%) |
| Ethnicity |  |  |  |
| Black | 207(1.28%) | 94(1.17%) | 113(1.4%) |
| White | 15259(94.61%) | 7636(94.69%) | 7623(94.53%) |
| BMI | 27.87(25.27-31.01) | 27.87(25.27-30.96) | 27.88(25.26-31.04) |
| Systolic blood pressure | 148(142-157) | 148(142-158) | 148(142-157) |
| Diastolic Blood Pressure | 87(80-93) | 87(80-93) | 87(80-93) |
| Glycated hemoglobin | 36(33.4-39) | 36(33.5-39) | 35.9(33.3-38.9) |
| Total cholesterol | 5.60(4.76-6.45) | 5.62(4.77-6.45) | 5.59(4.75-6.45) |
| HDL | 1.35(1.12-1.62) | 1.35(1.12-1.62) | 1.34(1.12-1.62) |
| LDL | 3.49(2.85-4.14) | 3.49(2.85-4.14) | 3.49(2.84-4.14) |
| eGFR | 92.87 (87.85-98.44) | 92.79 (87.93-98.39) | 92.92 (87.78-98.51) |
| Anti-hyperglycemic medication | 206(1.28%) | 99(1.23%) | 107(1.33%) |
| Anti-hypertensive medication | 4619(28.64%) | 2268(28.12%) | 2351(29.15%) |
| Currently smoking | 1559(9.67%) | 792(9.82%) | 767(9.51%) |
| HF | 1074(6.66%) | 541(6.71%) | 533(6.61%) |

Values are given as median (interquartile range), or n (%).

BMI, body mass index; HDL, high-density lipoprotein; LDL, low-density lipoprotein; eGFR, estimated glomerular filtration rate.

Mann–Whitney U for continuous variables, 𝜒2 for categorical variables; Benjamini–Hochberg correction for multiple testing.
