# Supplementary material for: Large-scale plasma proteomic profiling enhances prediction of heart failure among hypertensive individuals: prospective insights from the UK Biobank
Source: J Glob Health. 2026 Oct 19;16:04313. doi: 10.7189/jogh.16.04313 (PMC13688282; doi:10.7189/jogh.16.04313)
Supplement: Supplementary file 1 [file jogh-16-04313-s001.zip › jogh-16-04313-s007.docx]

Table S6 —Association between protein risk score of hypertension and the risk of incident HF

|  | **exposure** | **covariates** | **N/events** | **HR（95%Cl）** | **P** |
| --- | --- | --- | --- | --- | --- |
| Train cohort | Protein risk score  Model1  Model2 | Age，Sex  PCP-HF | 8064/541 | 3.06[2.84-3.30]  2.99[2.75,3.25] | 5.36*10^-191^  1.92*10^-146^ |
| Test cohort | Protein risk score  Model1 | Age，Sex | 8064/533 | 2.47[2.31-2.65] | 4.78*10^-143^ |
|  | Model2 | PCP-HF |  | 2.32[2.13,2.51] | 5.25*10^-93^ |
