# Supplementary material for: Large-scale plasma proteomic profiling enhances prediction of heart failure among hypertensive individuals: prospective insights from the UK Biobank
Source: J Glob Health. 2026 Oct 19;16:04313. doi: 10.7189/jogh.16.04313 (PMC13688282; doi:10.7189/jogh.16.04313)
Supplement: Supplementary file 1 [file jogh-16-04313-s001.zip › jogh-16-04313-s008.docx]

Supplementary Table 7 Internal model validation of training set: absolute discriminative capacities

| **Metric** | **Age & Sex** | **Age & Sex +**  **PRS** | **PCP-HF** | **PCP-HF +**  **PRS** |
| --- | --- | --- | --- | --- |
| Harrell’s C | 0.686 (0.665-0.707) | 0.827 (0.808-0.846) | 0.734 (0.712-0.755) | 0.830 (0.812-0.848) |
| Sensitivity | 0.756 (0.539-0.816) | 0.717 (0.628-0.766) | 0.689 (0.570-0.777) | 0.684 (0.620-0.800) |
| Specificity | 0.517 (0.475-0.741) | 0.778 (0.743-0.856) | 0.662 (0.570-0.778) | 0.806 (0.691-0.862) |

Model performance is assessed in the validation partition. Reported 95% confidence intervals (in brackets) are obtained via percentile distributions of 1000-fold bootstrap resampling on the validation partition.

PRS, Protein risk score; PCP-HF, Pooled Cohort Equations to Prevent Heart Failure.
