# Supplementary material for: Large-scale plasma proteomic profiling enhances prediction of heart failure among hypertensive individuals: prospective insights from the UK Biobank
Source: J Glob Health. 2026 Oct 19;16:04313. doi: 10.7189/jogh.16.04313 (PMC13688282; doi:10.7189/jogh.16.04313)
Supplement: Supplementary file 1 [file jogh-16-04313-s001.zip › jogh-16-04313-s009.docx]

Supplementary Table 8 Internal model validation of training set: metrics of relative performance

| **Metric** | **Age & Sex**  **vs.**  **Age & Sex + PRS** | **PCP-HF**  **vs.**  **PCP-HF + PRS** | **Age & Sex**  **vs.**  **PCP-HF** | **Age & Sex + PRS**  **vs.**  **PCP-HF** | **Age & Sex**  **vs.**  **PCP-HF + PRS** | **Age & Sex + PRS**  **vs.**  **PCP-HF + PRS** |
| --- | --- | --- | --- | --- | --- | --- |
| △C | 0.141 (0.119-0.163) | 0.096 (0.079-0.114) | 0.048 (0.034-0.063) | -0.093 (-0.112 , -0.075) | 0.144 (0.124-0.165) | 0.003 (0.000-0.006) |
| NRI | 0.461 (0.406-0.517) | 0.403 (0.341-0.461) | 0.232 (0.166-0.291) | -0.375 (-0.428 , -0.284) | 0.481(0.422-0.528) | 0.128 (0.048-0.221) |
| IDI | 0.131 (0.105-0.161) | 0.115 (0.089-0.144) | 0.020 (0.014-0.031) | -0.112 (-0.141 , -0.085) | 0.134(0.110-0.165) | 0.003 (-0.001-0.014) |

NRI, net reclassification improvement; IDI, integrated discrimination improvement; PCP-HF, Pooled Cohort Equations to Prevent Heart Failure.
